# Cigarette smoke condensate induces centrosome clustering in normal lung epithelial cells

**DOI:** 10.1002/cam4.5599

**Published:** 2023-01-09

**Authors:** Jose Thaiparambil, Chandra S. Amara, Subrata Sen, Nagireddy Putluri, Randa El‐Zein

**Affiliations:** ^1^ Houston Methodist Neal Cancer Center Houston Texas USA; ^2^ Department of Molecular and Cellular Biology Baylor College of Medicine Houston Texas USA; ^3^ Department of Translational Molecular Pathology UT MD Anderson Cancer Center Houston Texas USA

**Keywords:** carcinoma, centrosome clustering, mitotic spindle, mitosis, nonsmall‐cell lung

## Abstract

**Background:**

Unlike normal cells, cancer cells frequently have multiple centrosomes that can cluster to form bipolar mitotic spindles and allow for successful cell division. Inhibiting centrosome clustering, therefore, holds therapeutic promise to promote cancer cell‐specific cell death.

**Methods:**

We used confocal microscopy, real‐time PCR, siRNA knockdown, and western blot to analyze centrosome clustering and declustering using normal lung bronchial epithelial and nonsmall‐cell lung cancer (NSCLC) cell lines. Also, we used Ingenuity Pathway Analysis software to identify novel pathways associated with centrosome clustering.

**Results:**

In this study, we found that exposure to cigarette smoke condensate induces centrosome amplification and clustering in human lung epithelial cells. We observed a similar increase in centrosome amplification and clustering in unexposed NSCLC cell lines which may suggest a common underlying mechanism for lung carcinogenesis. We identified a cyclin D2‐mediated centrosome clustering pathway that involves a sonic hedgehog‐forkhead box protein M1 axis which is critical for mitosis. We also observed that cyclin D2 knockdown induced multipolar mitotic spindles that could eventually lead to cell death.

**Conclusions:**

Here we report a novel role of cyclin D2 in the regulation of centrosome clustering, which could allow the identification of tumors sensitive to cyclin D2 inhibitors. Our data reveal a pathway that can be targeted to inhibit centrosome clustering by interfering with the expression of cyclin D2‐associated genes.

## Graphical abstract

We performed Real‐Time PCR, siRNA knockdown, western blot, and confocal microscopy to analyze centrosome clustering and declustering using normal lung bronchial epithelial cells exposed to CSC and compared to non‐small cell lung cancer (NSCLC) cell lines. We identified a cyclin D2‐mediated centrosome clustering pathway that involves SHH‐FOXM1‐CCND2‐CDK2 axis which is critical for mitosis plays a key role in CSC induced Beas‐2B cells. This study suggests a novel role for cyclin D2 in the regulation of centrosome clustering and reveal a pathway that can be targeted to inhibit centrosome clustering by interfering with cyclin D2 associated genes.

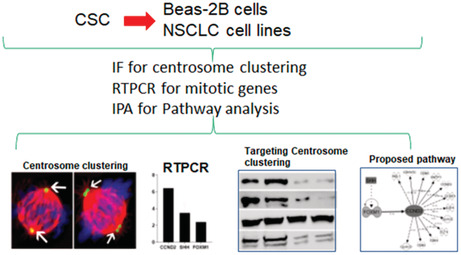

## 1 INTRODUCTION

Cigarette smoking is responsible for 90% of lung cancer cases. Cigarette smoke induces genomic instability through a well‐understood general mechanism, however, alterations in the centrosome dynamics are not known. The generation of more than two centrosomes, termed supernumerary centrosomes, is common in human cancers and correlates with chromosomal instability.^1^, ^2^, ^3^ Supernumerary centrosomes can cause cancer cells to die because of multipolar spindles and the resulting mitotic catastrophe and high levels of aneuploidy.^1^, ^4^ Alternatively, cancer cells can evade this process and form viable progeny by grouping their centrosomes into two polar groups, termed centrosome clustering, resulting in a functional pseudo‐bipolar structure.^1^, ^4^, ^5^, ^6^, ^7^, ^8^ Spindle formation time is prolonged in cells undergoing centrosome clustering. A pseudo‐bipolar spindle is often formed in these cells, with individual kinetochores attaching to microtubules coming from different poles, leading to lagging chromosomes during anaphase and playing a role in chromosomal instability.^7^, ^8^ Missegregation and aneuploidy in tumors with supernumerary centrosomes suggest that this process could increase mutation rates and thus contribute to cancer development and progression.^8^

Several studies have reported that inducing centrosome amplification increases tumor formation in *Drosophila melanogaster* ^9^ and mice.^10^, ^11^ Centrosomal abnormalities have been reported in prostate, breast, ovarian, pancreatic, and colon cancer^12^, ^13^, ^14^, ^15^ but have not been investigated in nonsmall‐cell lung cancer (NSCLC). Because supernumerary centrosomes are found almost exclusively in cancer cells, preventing their clustering during mitosis, and therefore inducing mitotic defects leading to mitotic catastrophe, could represent a novel approach to promote cell death of cancer cells but not normal cells.^1^

We have previously reported that two major carcinogens present in cigarette smoke, Benzo[a]pyrene (BaP) and 4‐(methylnitrosamino)‐1‐(3‐pyridyl)‐1‐butanone (NNK), perturb the mitotic spindle apparatus and induce centrosome amplification in human lung epithelial cells.^16^, ^17^ Since cigarette smoke contains a cocktail of chemical carcinogens that is not limited to NNK and BaP, in the current study, we investigate whether cigarette smoke exposure induces centrosomal abnormalities. While there are several reports of cigarette smoke‐induced DNA adducts in lung cancer, no studies have reported aberrant formation of centrosomal structure. In the current study, we hypothesized that cigarette smoke condensate (CSC) exposure induces centrosome clustering in normal lung epithelial cells allowing the identification of key molecular players involved in centrosome clustering and revealing a mechanistic link between cigarette smoke and lung cancer. Our results identified aberrant activation of the sonic hedgehog‐cyclin D2 (SHH‐CCND2) signaling axis and centrosome clustering in CSC‐exposed human bronchial epithelial cells. We obtained similar results in lung adeno‐ and squamous cell carcinoma cell lines not exposed to CSC. Analysis with real‐time‐polymerase chain reaction (RT‐PCR) showed a differential expression of genes in both normal and NSCLC cell lines. Finally, using Ingenuity Pathway Analysis software (Qiagen), we have identified a novel pathway associated with the SHH‐CCND2 axis that could play a role in centrosome clustering.

## 2 MATERIALS AND METHODS

### 2.1 Cell lines and culture

Beas‐2B human bronchial epithelial cells, two lung adenocarcinoma cell lines (H23 and HCC827), one squamous lung cancer cell line, H1703, and a large cell lung carcinoma cell line (H460) were obtained from ATCC® (catalog numbers: CRL‐9609, CRL‐5800, CRL‐2868, CRL‐5889, and HTB‐177). The lung adeno, squamous, and large cell carcinoma cell lines were maintained in an RPMI‐1640 medium according to the recommended protocol. Beas‐2B cells were cultured as described in our previous study.^11^ Briefly, the flasks, plates, and dishes for Beas‐2B culture were coated with 0.03 mg/ml PureCol (Sigma®), 0.01 mg/ml fibronectin (Sigma®), and 0.01 mg/ml bovine serum albumin (Sigma®) dissolved in BEBM for at least 2 h at 37°C, which was vacuum aspirated to dry. A BEGM‐completed growth medium containing the same concentrations of PureCol, fibronectin, and bovine serum albumin was used for culture of the cells at 37°C in 95% air and 5% CO_2_ atmosphere, which were incubated until reaching 80% confluence.

### 2.2 XTT cell proliferation assay

Beas‐2B cells were seeded at 1 × 10^4^ cells/well in a 96‐well plate. Different percentages of CSC (10%, 20%, 40%, 60%, 80%, and 100%) were added to the medium for 72 h. Next, 50 μl of XTT labeling mixture was added to each well (purchased from ATCC®, Cat. no. 30‐1011K). Optical density was measured in triplicate wells at 490 nm with a reference wavelength of 650 nm using a Biotech® microplate reader.

### 2.3 Preparation of CSC media

**FIGURE 1:**
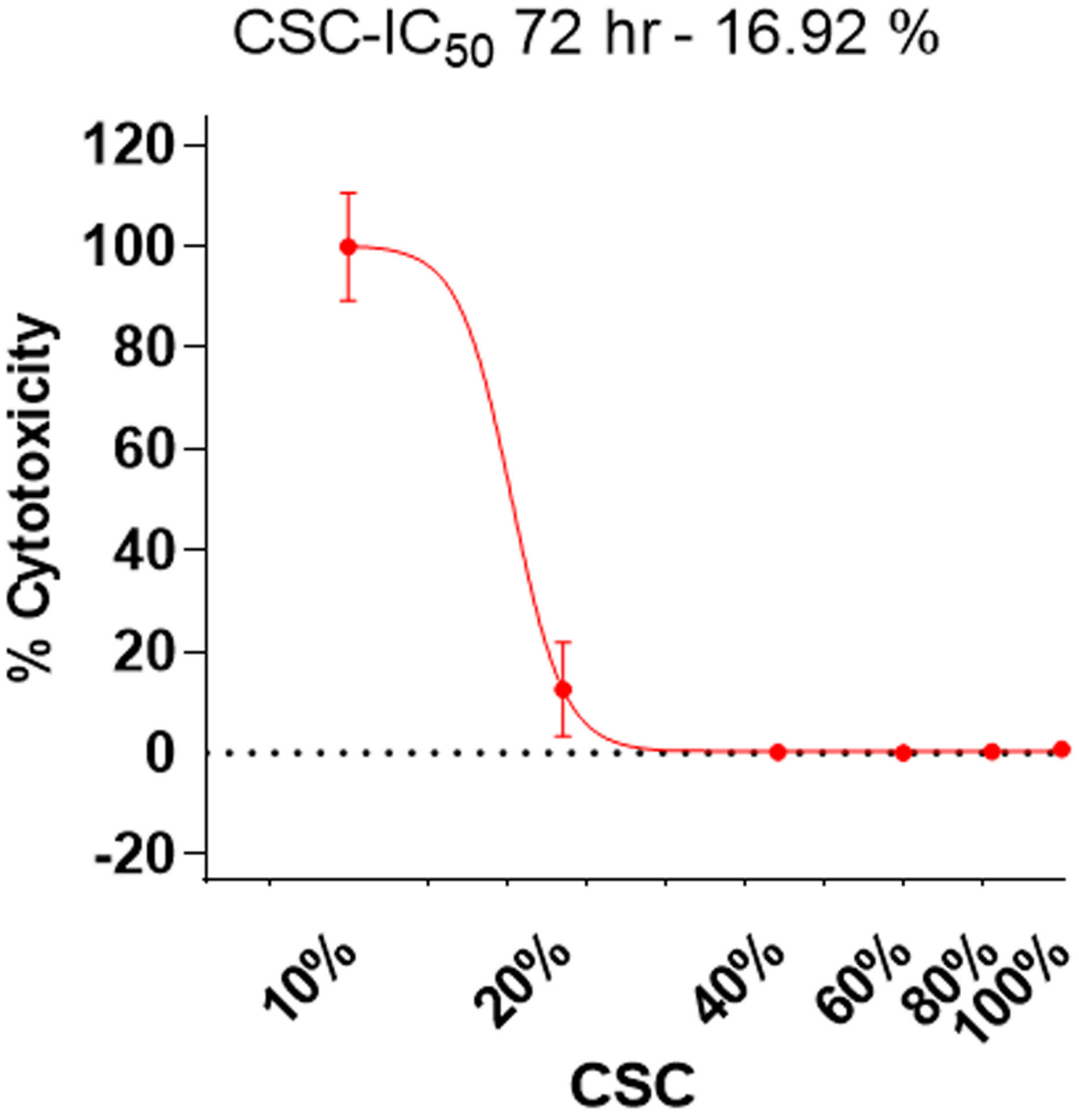
Determination of the IC_50_ of CSC in Beas‐2B cells. Cytotoxicity was analyzed in Beas‐2B cells treated with different percentages of CSC (10%, 20%, 40%, 60%, 80%, and 100%) for 72 h. GraphPad prism software was used to calculate the IC_50_ (16.9%). We selected 10% CSC for further experiments which showed more than 90% of cell viability in order to provide an accurate evaluation of mitotic alterations.

In the smoke extraction preparation, cigarettes were burned for 4–5 min/cigarette to mimic smoke exposure. For the preparation of CSC media, a burned cigarette was bubbled through a 100 ml BEBM medium in a special chamber. The resulting medium was termed 100% CSC cell culture media. After obtaining results from the XTT assay where cell viability with 10% CSC was 90% (Figure 1), we used 10% CSC for all subsequent experiments. Beas‐2B cells were the only cells exposed to CSC.

### 2.4 Preparation of cells for immunofluorescence

Beas‐2B and NSCLC cells were synchronized in mitosis using a double thymidine block as detailed in our previous study.^17^ Briefly, cells were treated with 2.5 mM thymidine (Cat# T1895, Sigma–Aldrich) for 24 h, incubated in a medium without thymidine for 14 h, and incubated with 2.5 mM thymidine for another 24 h, all at 37°C. After washing cells with PBS, they were then allowed to grow for 72 h. The mitotic abnormalities (multipolar spindles) were examined and compared between CSC‐treated cells and untreated (control) cells.

### 2.5 Spindle visualization via immunofluorescence

Immunofluorescent staining was conducted using standard protocols.^18^ Briefly, cells were incubated on no. 1.5 coverslips in tissue culture plates to adhere overnight. PHEMO buffer, which consisted of 3.7% formaldehyde, 60 mM piperazine‐*N*,*N*′‐bis(2‐ethanesulfonic acid), 25 mM HEPES, 0.05% glutaraldehyde, 10 mM EGTA, and 4 mM MgSO_4_, was used to fix cells for 10 min. After washing in PBS, cells were blocked with 2.5% normal goat serum for 15 min, then incubated overnight with primary antibody (1:1000) at 4°C. After washing with PBS, cells were incubated with 1:500 Alexa Fluor‐conjugated secondary antibody at room temperature for 2 h. For each pair of primary–secondary antibodies, the same steps were sequentially repeated.

Primary antibodies used include Rabbit polyclonal antipericentrin (Abcam® Cat. no. ab 4448) and mouse monoclonal anti‐α‐Tubulin (Sigma® Cat. no. T‐9026). Secondary antibodies used include Alexa Fluor 488 or 555 (Invitrogen®). Nuclei were stained by incubating cells with mounting media containing DAPI (4′,6‐diamidino‐2‐phenylindole; Vectashield®).

### 2.6 Confocal microscopy

Fluorescence imaging was conducted on the Nikon A1R® confocal imaging system equipped with a Nikon® oil immersion Plan‐APO 60x numerical aperture 1.40 lens. The Nikon® NIS Elements software was used for imaging control and projections. With acquisition settings kept constant, Z‐stacks of images at 0.2‐mm intervals were acquired, and maximum intensity projections were created.^16^, ^17^, ^18^, ^19^

### 2.7 Measuring centrosome clustering

As centrosome clustering may be very tight, counting exact centrosome numbers in each cluster is difficult. An acceptable alternative for measuring centrosome clustering is measuring the volume of each centrosome cluster using a published protocol.^19^ One hundred mitotic cells were scored to determine the percentage with centrosome clustering, and a two‐tailed Fisher exact test was used to determine significant differences between CSC‐treated and untreated experimental conditions using GraphPad Prism software.

### 2.8 Measuring misaligned or lagging chromosomes

Metaphase, anaphase, and telophase cells were scored for the presence or absence of lagging chromosomes and bridges (as we previously detailed in 19, 20). A total of 50 cells were scored to determine the chromosome segregation errors and a two‐tailed Fisher exact test was used to analyze group differences.

### 2.9 Real‐time‐polymerase chain reaction (RT‐PCR)

To identify genes associated with centrosome clustering, we used a custom‐designed plate with mitotic spindle‐associated genes^18^ selected based on our previously published study (Figure S1).^17^ The RNeasy plus mini kit (Qiagen® Cat# 74104) was used to extract total RNA from Beas‐2B and NSCLC cell lines according to the manufacturer's instructions. The iScript cDNA synthesis kit (Bio‐Rad Cat# 1708841) was used for reverse transcription, using 500 ng RNA and SSO advanced Universal SYBR® Green Supermix (Cat# 172‐5271). The CFX96 Touch RT‐PCR Detection System (Bio‐Rad®) was used with the following cycle: 95°C for 10 min (1 cycle), 95°C for 15 s, 60°C for 1 min, 95°C for 15 s for 40 cycles followed by 95°C for 15 s, and 60°C for 1 min. PCR product specificity was confirmed by melt curve analysis. Data analysis was performed using CFX Manager Software (Bio‐Rad®). Data were normalized that of actin and GAPDH from the same sample, and fold changes in gene expression were calculated using the ΔΔCt method.

### 2.10 Western blot analysis

To assess protein expression, western blot analysis was conducted for selected genes identified in our RT‐PCR analysis. Equal amounts of protein (50 μg) from each cell lysate sample were separated by SDS‐PAGE and transferred onto a PVDF membrane by electroblotting (Bio‐Rad). All primary and secondary antibodies used in the western blot analysis were purchased from Abcam®. Membranes were exposed to X‐ray film after incubation with enhanced chemiluminescence. Western blots shown are representative of at least three replicates.

### 2.11 RNAi gene silencing

To silence CCND2, SHH, and FOXM1, Beas‐2B cells were transiently transfected with SMARTpool siRNA (Dharmacon®, Thermo Scientific) using the transfection reagent Lipofectamine according to the manufacturer's instructions (Thermo Fisher Scientific®, Cat# 52887). siRNAs‐targeting CCND2 (E‐003211‐00‐0020), SHH (E‐006036‐00‐0020), and FOXM1 (L‐009762‐00‐0020) as well as nonspecific control siRNA were used.

### 2.12 Ingenuity pathway analysis

We used ingenuity pathway analysis (IPA) network analysis (Qiagen) based on our previously published study^17^, ^20^ to identify the pathway that may control centrosome clustering. Two scores were used: the enrichment score assesses the extent of overlap between the observed and predicted gene sets (Fisher's exact test *p* value). The *z*‐score measures how well the observed and predicted patterns of up‐ and downregulation match.

### 2.13 Statistical analysis

Prism 7 (GraphPad® Software Inc.) was used for statistical analyses. CSC‐treated cells, untreated control cells, and NSCLC (adeno and squamous cell carcinoma) cell lines were compared using two‐way ANOVA analysis. The significance of differences between cell lines was assessed with Bonferroni's multiple comparison test, with differences considered significant with a *p* value of <0.05.

## 3 RESULTS

### 3.1 Cytotoxicity of CSC on normal lung epithelial cells

We have previously reported that BaP and NNK induce mitotic abnormalities such as centrosome amplification, misoriented spindles, and misaligned chromosomes at metaphase.^16^, ^17^ Based on these studies, we treated lung broncho‐epithelial cells (Beas‐2B) with CSC to mimic exposure to the cocktail of chemicals found in cigarette smoke. We used the XTT cytotoxicity assay for Beas‐2B cells treated with media containing different percentages of CSC to calculate the IC_50_ value. Figure 1 shows an IC_50_ of 16.9%, with over 90% cell viability at 10% CSC concentration thus allowing for the accurate evaluation of mitotic alterations.

### 3.2 Effects of CSC on centrosome clustering

**FIGURE 2:**
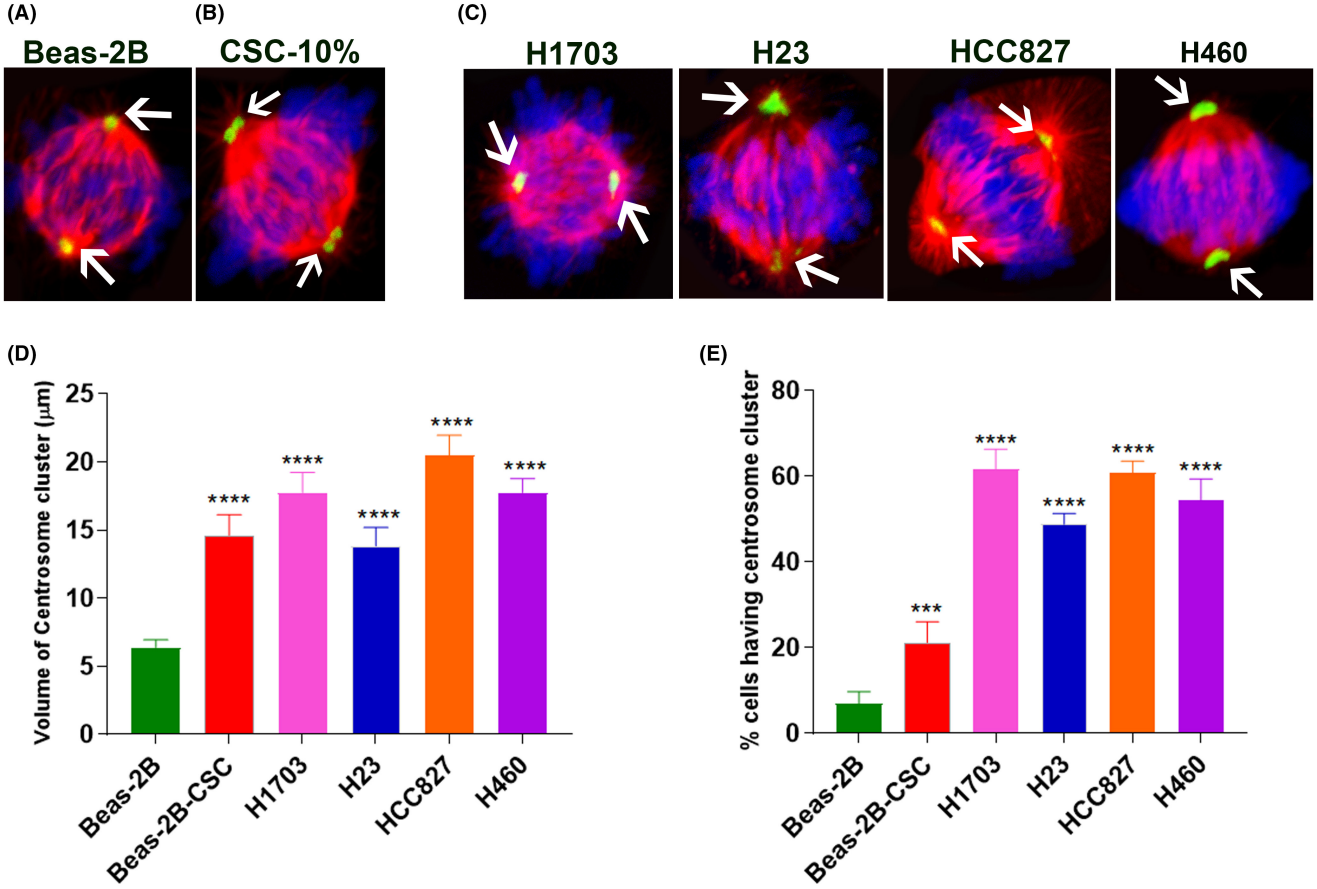
Centrosome clustering and centrosome volume in CSC exposed and untreated Beas‐2B with three NSCLC cell lines. Immunofluorescence microscopy was used to visualize Beas‐2B cells stained for tubulin (red; spindle), pericentrin (green; centrosomes), and DNA (DAPI‐blue). Scale bar = 10 μm. (A) Control mitotic cell (no CSC exposure) shows two centrosomes (white arrows) at opposite poles that lead to bipolar spindles that organize chromosomes along the metaphase plate, (B) CSC exposed mitotic cell showing clustered centrosomes (white arrows) on both poles, (C) four NSCLC cell lines, H1703, H23, HCC827, and H460 show clustered centrosomes (White arrows, pericentrin) similar to CSC‐exposed Beas‐2B cells in contrast to unexposed Beas‐2B cells, (D) differences in centrosome volume in the unexposed and CSC‐exposed Beas‐2B cells compared with three NSCLC cell lines. Centrosome volume in CSC‐exposed Beas‐2B cells was significantly increased compared with unexposed Beas‐2B cells (*p* < 0.0001). Similarly, all four NSCLC cell lines exhibited a significant increase in the centrosome volume compared with unexposed Beas‐2B cells. The data shown represent the mean ± SD from three independent experiments with 50 total cells scored, (E) significantly increased percentage of centrosome clustering in CSC exposed and unexposed Beas‐2B cells compared with four NSCLC cell lines. The extent of centrosome clustering was significantly higher in CSC exposed and NSCLC cell lines compared with unexposed Beas‐2B cells (*p* < 0.0001). The data shown represent the mean ± SD from three independent experiments with 50 cells scored.

We focused on centrosome clustering, based on our previous studies showing that centrosome amplification was one of the major mitotic abnormalities associated with BaP and NNK^16^, ^17^ as well as recent evidence from in vitro and in vivo models showing a strong correlation of centrosome clustering with tumor recurrence and metastasis.^21^, ^22^ We assessed whether mitotic centrosome clusters are induced in Beas‐2B cells exposed to CSC, compared to vehicle‐treated control. The centrosomes in CSC‐treated cells (Figure 2B) appeared significantly larger than centrosomes in the vehicle‐treated control (Figure 2A) (CSC >20 vs. 7 for control, *p* < 0.0001). We determined centrosomes volumes by measuring pericentrin intensity using Nikon Elements 5.1 software according to our previously published procedure (Figure 2D).^19^ If the volume of a centrosome was above the maximum observed in vehicle‐treated Beas‐2B cells (6.3 μm^3^), it was considered centrosome amplification. CSC‐exposed Beas‐2B cells showed an increased volume of centrosome compared with vehicle‐treated control (Figure 2D). This result was well correlated with the amplification of centrosome cluster in 20% of cells (two‐way ANOVA, *p* < 0.0002) in response to CSC (Figure 2E). We compared CSC‐induced centrosome volume and clustering in Beas‐2B cells with lung squamous cell carcinoma (H1703) and adenocarcinoma (H23 and HCC827) and a large cell carcinoma cell lines and observed increased centrosome volume (>15 μm, *p* < 0.0001, Figure 2D) and clustering (50%–60% cells, two‐way ANOVA, *p* < 0.0001) (Figure 2E) in cancer cell lines. This suggests that CSC‐induced centrosome clusters in Beas‐2B cells were similar to those in adeno and squamous cell carcinoma cells and that identifying the molecular players responsible for this centrosome clustering could uncover novel pathways that can be therapeutically targeted.

### 3.3 Overexpression of similar genes in CSC‐treated lung epithelial cells and NSCLC

**FIGURE 3:**
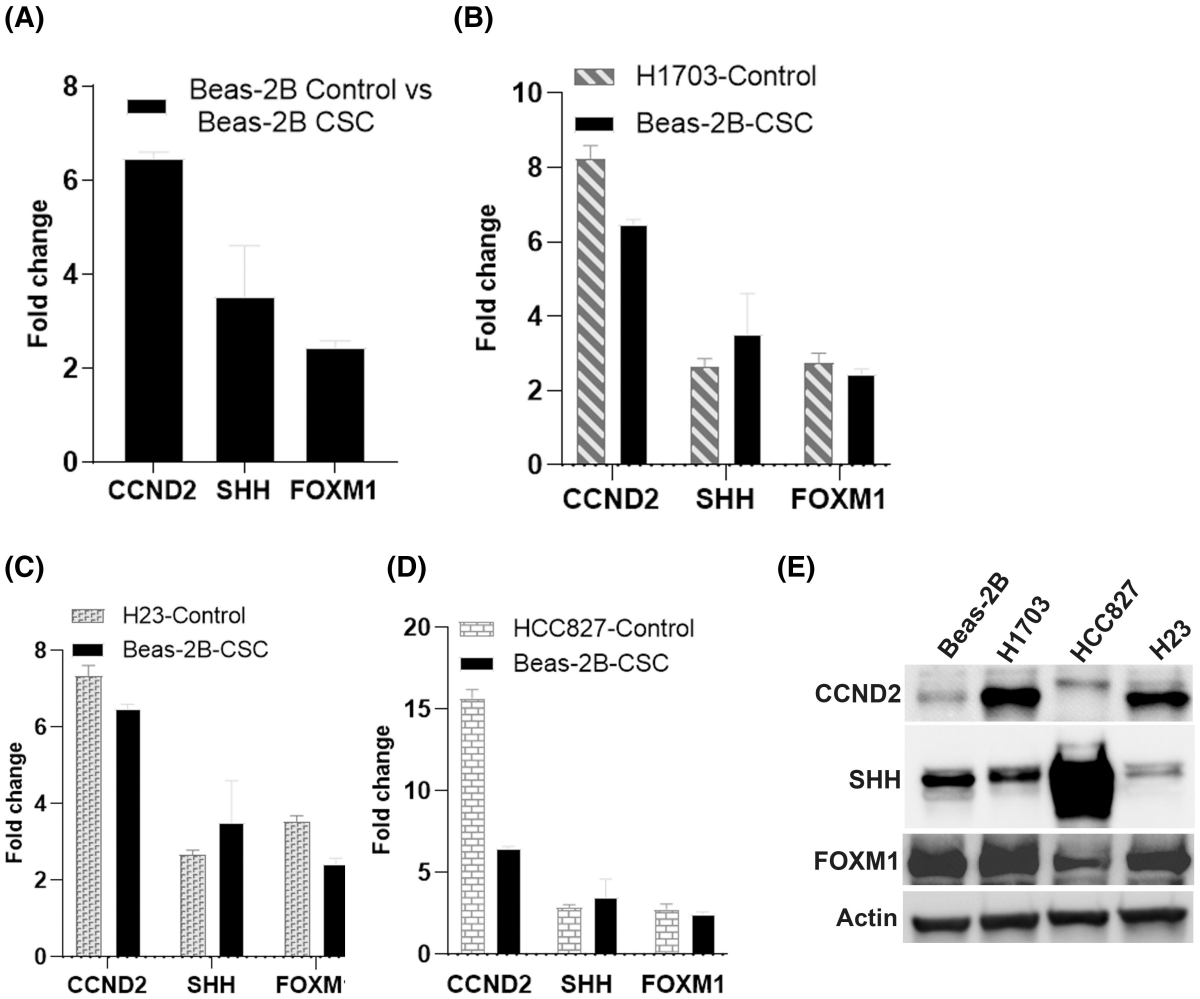
Effect of CSC on gene expression. Beas‐2B cells were treated with CSC for 72 h and compared with untreated Beas‐2B cells and four NSCLC cell lines (H1703, H23, HCC827, and H460). (A) Unique expression of CCND2, SHH, and FOXM1 (>2 fold) in CSC exposed and (B–D) NSCLC cell lines in contrast to untreated Beas‐2B cells. CCND2 expression increased more than six folds in CSC exposed and NSCLC cell lines compared with untreated Beas‐2B cells. Upstream targets of CCND2, such as SHH and FOXM1, also showed a more than a two‐fold increase in CSC exposed and NSCLC cell lines (A–D) compared with unexposed Beas‐2B cells. Western blot (E) shows increased protein expression of CCND2 in three NSCLC cell lines (HCC827, compared with untreated Beas‐2B cells). Increased protein expression of SHH and FOXM1 was predominantly observed in H1703 and HCC827, respectively (E).

Since similar genes were upregulated in CSC‐treated Beas‐2B and vehicle‐treated human NSCLC cell lines (two adenocarcinomas and one squamous cell carcinoma), we analyzed the expression of 20 mitotic genes by RT‐PCR analysis in these cell lines to identify genes that play a key role in centrosome clustering. We observed a marked increase of CCND2, FOXM1, and SHH (>2‐fold) in both CSC‐treated Beas‐2B and NSCLC cell lines (Figure 3A–D). The remaining genes showed common expression patterns in all cell lines (Figure S2). CCND2 expression was increased in all cell lines with a higher increase in cancer cell lines (>7–15 fold) compared with CSC‐treated Beas‐2B cells (>5–6 fold) (Figure 3A–D). This observation was further confirmed by western blot analysis (Figure 3E). Increased expression of FOXM1 and SHH proteins was predominantly observed in H1703 and HCC827 cell lines, respectively, in contrast to untreated Beas‐2B cells. CCND2 was the only common gene significantly upregulated in NSCLC and CSC‐treated Beas‐2B cell lines, suggesting a role of CCND2 in promoting centrosome clustering to help cancer cells divide and continue generating progeny.

### 3.4 Targeting centrosome clustering to inhibit normal cell division

**FIGURE 4:**
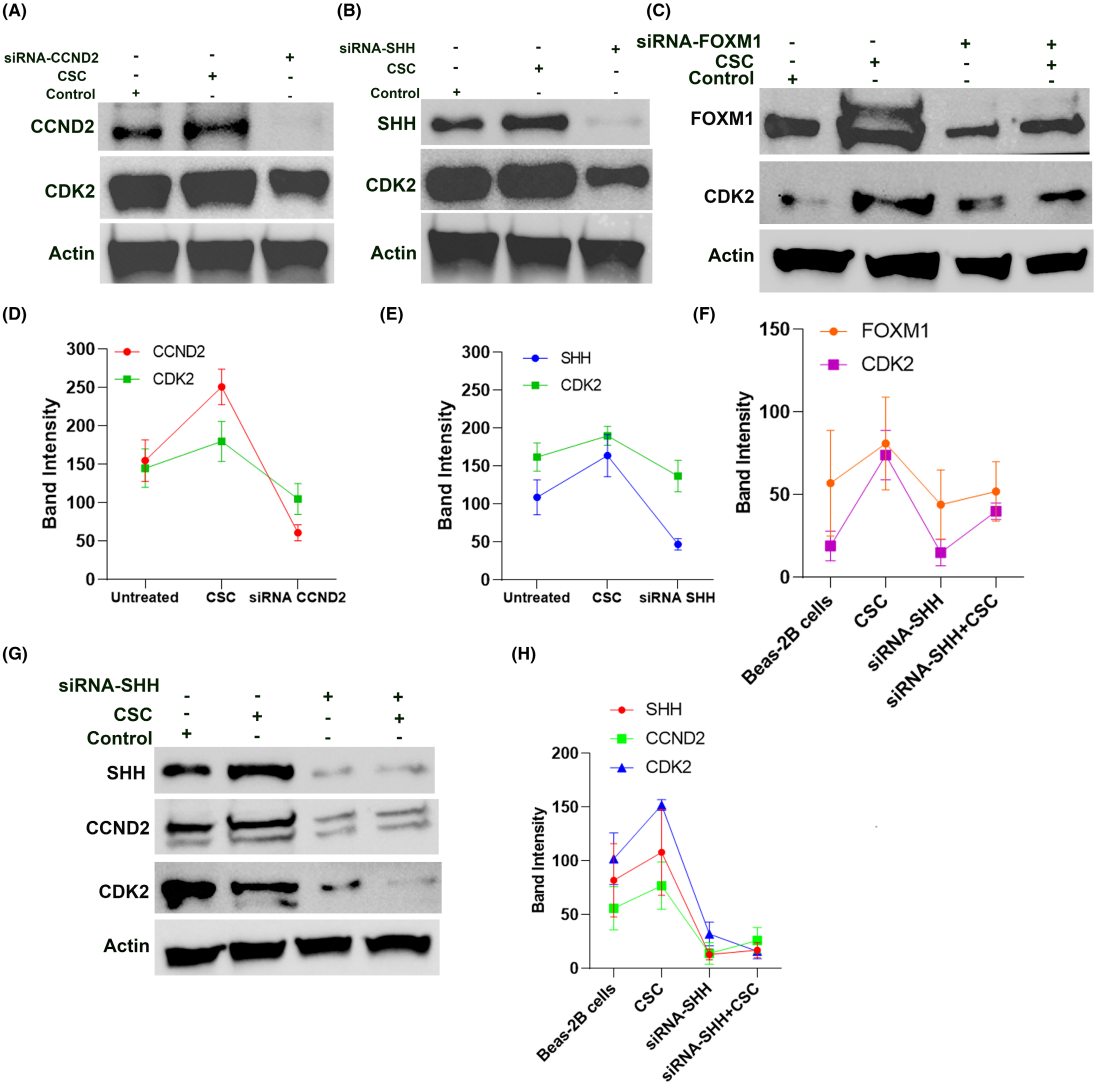
Effect of CCND2 knockdown in inducing declustering of centrosomes. (A) CCND2 expression in unexposed, CSC exposed, and siRNA knockdown Beas‐2B cells. CSC‐exposed Beas‐2B cells showed a higher expression of CCND2 compared with untreated and siRNA knockdown cells (A, D). Knockdown of CCND2 showed a corresponding decrease of CDK2 in Beas‐2B cells (A, D). Similarly, B, E and C, F show a higher increase of SHH and FOXM1 in CSC‐exposed Beas‐2B compared with untreated and siRNA knockdown Beas‐2B cells alone and with CSC, respectively. SHH and FOXM1 knockdown also decreased CDK2 expression as shown in B, E and C, F. (G, H) The suppression of SHH, CCND2, and CDK2 in SHH knockdown Beas‐2B cells in the presence or absence of CSC compared with CSC‐exposed Beas‐2B cells.

**FIGURE 5:**
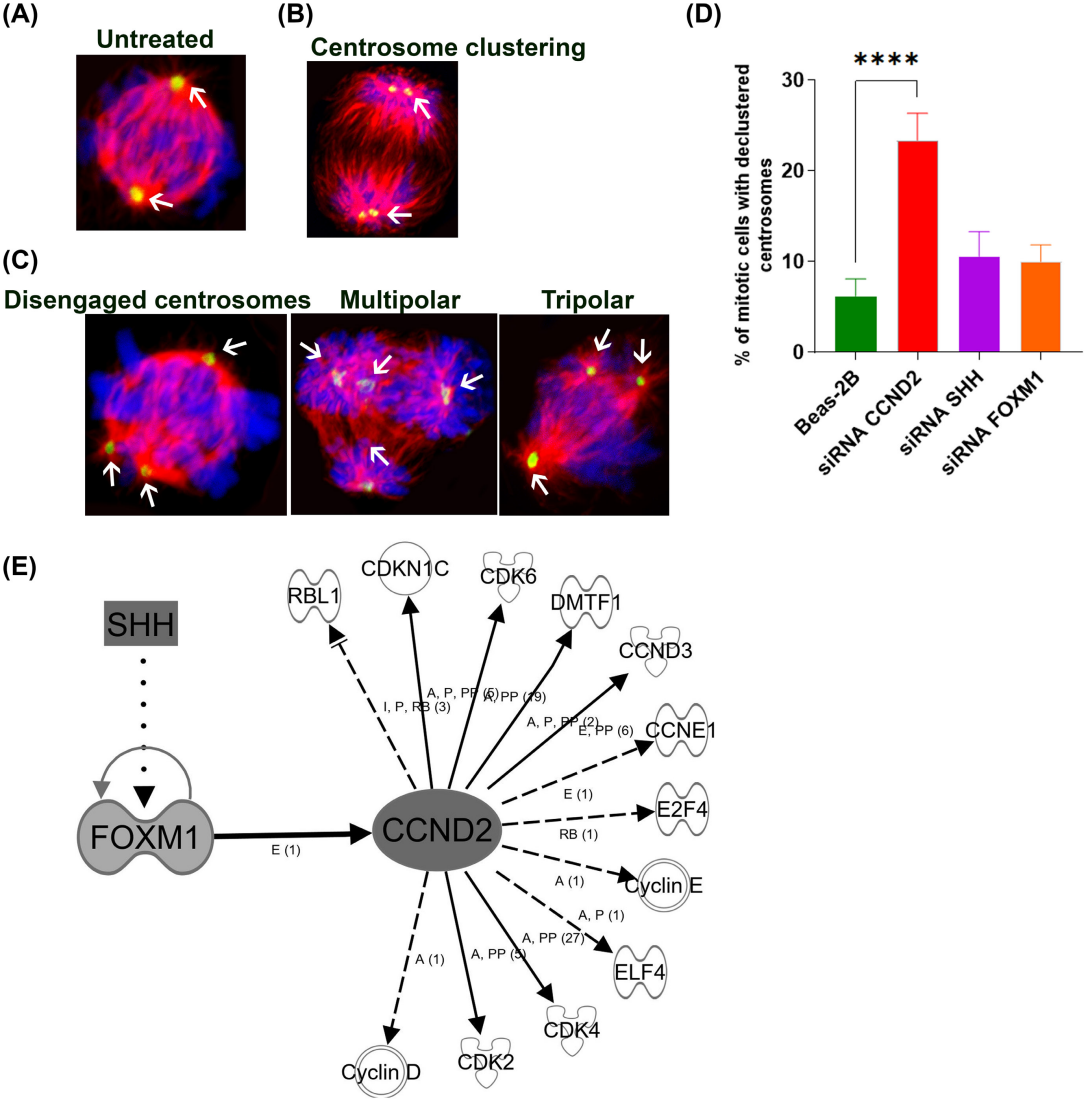
Effect of CCND2 knockdown and proposed pathway for regulation of centrosome clustering in normal lung broncho epithelial cells. Representative immunofluorescence confocal micrographs showing normal centrosomes, (A) Clustered centrosomes in unexposed and CSC‐exposed Beas‐2B cells (B) showing normal centrosomes and centrosome clusters in metaphase cells (white arrows) visualized by immunostaining for pericentrin (green; centrosomes) and *α*‐tubulin (red; microtubules), and DAPI staining (blue; DNA). (C) CCND2 knockdown induced declustered centrosomes that led to the formation of disengaged centrosomes (white arrows), multipolar, and tripolar spindles (white arrows). (D) Bar graphs representing the percentage of cells showing centrosome declustering in CCND2, SHH, and FOXM1 siRNA knockdown in Beas‐2B cells compared with untreated cells. The CCND2 knockdown induced a significant increase of disengaged, multipolar, and tripolar spindles in contrast to the knockdown of SHH and FOXM1 (*p* < 0.0001). The declustered centrosomes were scored from randomly selected fields totaling 50 cells per sample. Scale bar: 5 μm. (E) CCND2 is an intermediate regulator that directly activates CDKs (solid arrows) to induce centrosome clustering in CSC‐exposed and NSCLC cell lines and siRNA interference with CCND2 inhibits the activity of CDKs and induce centrosome declustering that could lead to multipolar spindles. SHH and FOXM1 act as upstream regulators of CCND2 and interfering with siRNA also inhibited CDKs.

As we observed CCND2, SHH, and FOXM1 overexpression in response to CSC and a similar or higher level in NSCLC cancer cell lines, we, therefore, treated Beas‐2B cells with short interfering RNAs (siRNAs) for all the three genes in order to determine the effect of silencing these genes in centrosome clustering. The siRNA knockdown was confirmed by western blot analysis (Figure 4A–F). We found that following CCND2 knockdown, the protein expression of CDK2 (downstream targets of CCND2) was reduced (Figure 4A,D). Similarly, SHH and FOXM1 (upstream targets of CCND2) knockdown also reduced the expression of CDK2 (Figure 4B,E and C,F), suggesting that CCND2 could be another potential drug target to inhibit the activity of CDKs. To further explore whether SHH regulates CCND2, we assessed the expression of CCND2 and CDK2 in Beas‐2B cells after knocking down SHH and found that both CCND2 and CDK2 expression were suppressed in the presence or absence of CSC. Furthermore, knockdown of CCND2 induced declustering of centrosomes as evidenced by the formation of disengaged centrosomes in metaphase or multipolar or tripolar spindles (Figure 5C) compared with CSC‐exposed (Figure 5B) and untreated Beas‐2B cells (Figure 5A). Silencing of CCND2 showed a marked increase in centrosome declustering compared with silencing the expression of SHH and FOXM1 as shown in Figure 5D (*p* < 0.0001). This suggests that interfering with CCND2 could disrupt the centrosome clustering mechanism.

### 3.5 Novel pathway that targets centrosome clustering

We observed CCND2 overexpression in both CSC‐treated Beas‐2B cells and NSCLC cell lines, and studies have shown overexpression of CCND2 in gastric cancer that is associated with tumor progression and metastasis.^20^ We identified genes upstream and downstream to CCND2 by Ingenuity Pathway Analysis using two scores, as described in our previous study^17^: one that measures how well the observed regulated gene sets match those predicted and another that measures how observed up‐ and downregulation patterns with those predicted. Our data unraveled a pathway that contains 15 total genes including CCND2, 12 downstream targets that interact directly or indirectly with CCND2, and 2 upstream FOXM1 and SHH as shown in Figure 5E.

## 4 DISCUSSION

In the present study, we report centrosome clustering in human lung epithelial cells in response to cigarette smoke, for the first time to our knowledge. We have identified a pathway involving SHH‐FOXM1‐CCND2 which was elevated in response to exposure to CSC, and the genes associated with this pathway could play a role in centrosome clustering in NSCLC cell lines. Our previous studies showed that NNK and BaP, two major carcinogens present in cigarette smoke, perturb the mitotic spindle apparatus and induce centrosome amplification in lung epithelial cells.^16^, ^17^ In this study, Beas‐2B‐treated cells with CSC showed aberrant activation of the SHH‐FOXM1‐CCND2 signaling axis and centrosome clustering. SHH is one of the predominant ligands in the hedgehog pathway and was reported to contribute to airway inflammation induced by cigarette smoke via inflammatory cytokine regulation in A549 cells.^21^ Our findings are supported by a recent study reporting that mice exposed to cigarette smoke showed a higher expression of SHH.^22^ Transcription factor FOXM1 plays a multifaceted role in pulmonary disease^23^, ^24^, ^25^, ^26^ and is required for both the G1/S and G2/M transitions as well as M phase progression.^26^ We observed increased FOXM1 and SHH RNA expression in NSCLC cells and CSC‐treated Beas‐2B cells, even though the protein expression of both were predominant in H1703 (squamous cell carcinoma) and HCC827 (adenocarcinoma) cell lines, respectively. Consistent with this, a recent study reported high FOXM1 expression in NSCLC tissues and cell lines.^22^ CCND2 is overexpressed in gastric, ovarian, and testicular cancer^20^, ^27^, ^28^ and its overexpression in NSCLC tumor formation is not completely understood despite a report showing that miR‐4317 suppresses NSCLC growth through inhibition of CCND2.^29^ In our study, CCND2 expression in CSC‐treated Beas‐2B cells correlated with a higher fold increase in all the NSCLC cell lines tested, suggesting a possible pathway for lung tumorigenesis through induction of centrosome clustering.

The presence of multiple centrosomes in a cell, known as centrosome amplification, has been accepted as a hallmark of cancer. These supernumerary centrosomes can cause a mitotic catastrophe,^4^ but some cancer cells group these centrosomes into two clusters that allow cell cycle progression and the production of viable progeny.^8^ We observed centrosome amplification in Beas‐2B cells in response to CSC that is comparable to that seen in NSCLC cell lines, therefore, inhibition of centrosome clustering may serve as a potential strategy to push cancer cells toward mitotic catastrophe and subsequent cell death. As previous reports have suggested that cells with centrosome amplification have metastatic potential, one potential mechanism to suppress metastasis is antagonizing centrosome clustering. CCND2 inhibition prevents the proliferation and metastasis of NSCLC.^30^, ^31^ In our study, inhibiting CCND2 expression induced centrosome declustering as evidenced by the formation of disengaged centrosomes and multipolar spindle apparatus (Figure 4I), suggesting a new mechanism by which CCND2 inhibitors may therapeutically target NSCLC cells. We envision that inhibiting CCND2 would derail centrosome clustering and cause spindle multipolarity (as shown in Figure 4I), leading to nonviable progeny. Because centrosome clustering occurs in metaphase, disrupting the cluster might interfere with cell division, revealing a novel therapeutic approach.

In addition, we also observed other genes that were differently expressed in NSCLC cells (Figure S2). We identified a pathway containing 12 downstream genes that directly or indirectly interact with CCND2 and could potentially serve as a therapeutic target for NSCLC (Figure 5). Among 12 genes, a few CDKs (CDK2, CDK4, and CDK6) are downstream of the CCND2 pathway and are critical for cell cycle regulation, suggesting this pathway could be targeted in future treatment approaches. A series of preclinical and clinical studies have been conducted with CDK4/6 inhibitors in NSCLC with promising results.^32^ It has recently been shown that CDK2 is targetable, and seliciclib (CYC202), a CDK2/7/9 inhibitor, induces apoptosis in lung cancer cells with supernumerary centrosomes through multipolar anaphase, which is termed anaphase catastrophe.^33^

Disrupting the clustering of centrosomes is thought to have great potential as a therapeutic strategy.^34^, ^35^ However, the signaling pathway that regulates centrosome clustering and its function in cancer therapy remain largely unknown. A previous study^35^ showed that in the context of DNA damage stress, such as oxidative stress induced by cigarette smoke, centrosome clustering can lead to therapeutic resistance and tumor recurrence.^36^ Centrosome declustering drugs (such as Griseofulvin, Reduced‐9‐bromonoscapine, and PJ‐34) have been reported to scatter centrosomes leading to increased spindle multipolarity and therefore metaphase catastrophe.^37^, ^38^ This suggests that treatment with centrosome de‐clustering drugs, preventing centrosome clustering in NSCLC cell lines, could be a viable option for inhibiting the proliferation of NSCLC cells.

This study shows for the first time a CSC‐induced centrosome clustering pathway representing a novel mechanism by which cancer cells may progress through the cell cycle and survive with continued proliferation. CCND2 overexpression may have a key role in the development of NSCLC and inhibiting CCND2 could be a novel therapeutic strategy to suppress the proliferation and survival of lung tumor. Previous studies show that CDK2 is the major conductor of the centrosome cycle and that it mediates centrosome amplification.^39^ In addition, CDKs, downstream targets of CCND2, may play a major role in lung carcinogenesis, and developing CCND2 inhibitors that suppress CDKs may also have therapeutic potential. Future studies on CCND2 amplification in lung tumor biopsies and tissues are warranted to investigate if patients whose tumors contain cells undergoing centrosome amplification would benefit from treatment with CCND2 inhibitors.

## AUTHOR CONTRIBUTIONS

**Jose Thaiparambil:** Conceptualization (lead); data curation (lead); formal analysis (lead); investigation (lead); methodology (lead); project administration (lead); resources (lead); software (lead); supervision (lead); validation (lead); visualization (lead); writing – original draft (lead); writing – review and editing (lead). **Chandra Sekhar Amara:** Methodology (supporting); writing – review and editing (supporting). **Subrata Sen:** Methodology (supporting); writing – review and editing (supporting). **Nagireddy Putluri:** Methodology (supporting); writing – review and editing (supporting). **Randa El‐Zein:** Conceptualization (lead); data curation (lead); formal analysis (lead); funding acquisition (lead); investigation (lead); project administration (lead); resources (lead); supervision (lead); visualization (lead); writing – review and editing (lead).

## FUNDING INFORMATION

This research was supported by the NIH/NCI U01CA189240 (REZ), NIH/NCI R01CA216426 (N.P./REZ), NIH/NCI R01CA220297 (N.P), DOD W81XWH‐21‐1‐0613 (N.P), P30ES030285, and P42ES027725.

## CONFLICT OF INTEREST

The authors declare that they have no conflict of interest.

## ETHICAL STATEMENT

No human or animal studies are presented in this manuscript. No potentially identifiable human images or data are included in this study.

## Supporting information


Figure S1

Figure S2

## Data Availability

The data related to this article are available upon reasonable request to a corresponding author.
