# Supplementary figures and images for: Cigarette smoke condensate induces centrosome clustering in normal lung epithelial cells
Source: Cancer Med. 2023 Jan 9;12(7):8499–509. doi: 10.1002/cam4.5599 (PMC10134322; doi:10.1002/cam4.5599)

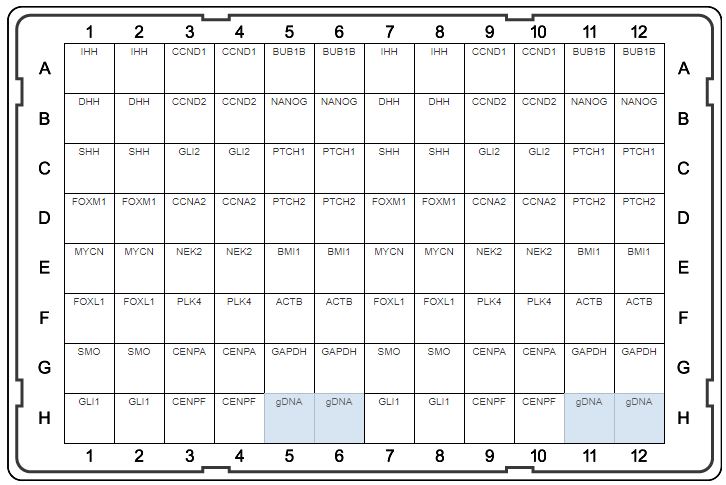

Supplement: Supplementary file 1 — Figure S1 [file CAM4-12-8499-s002.JPG]

Supple Figure-2

A

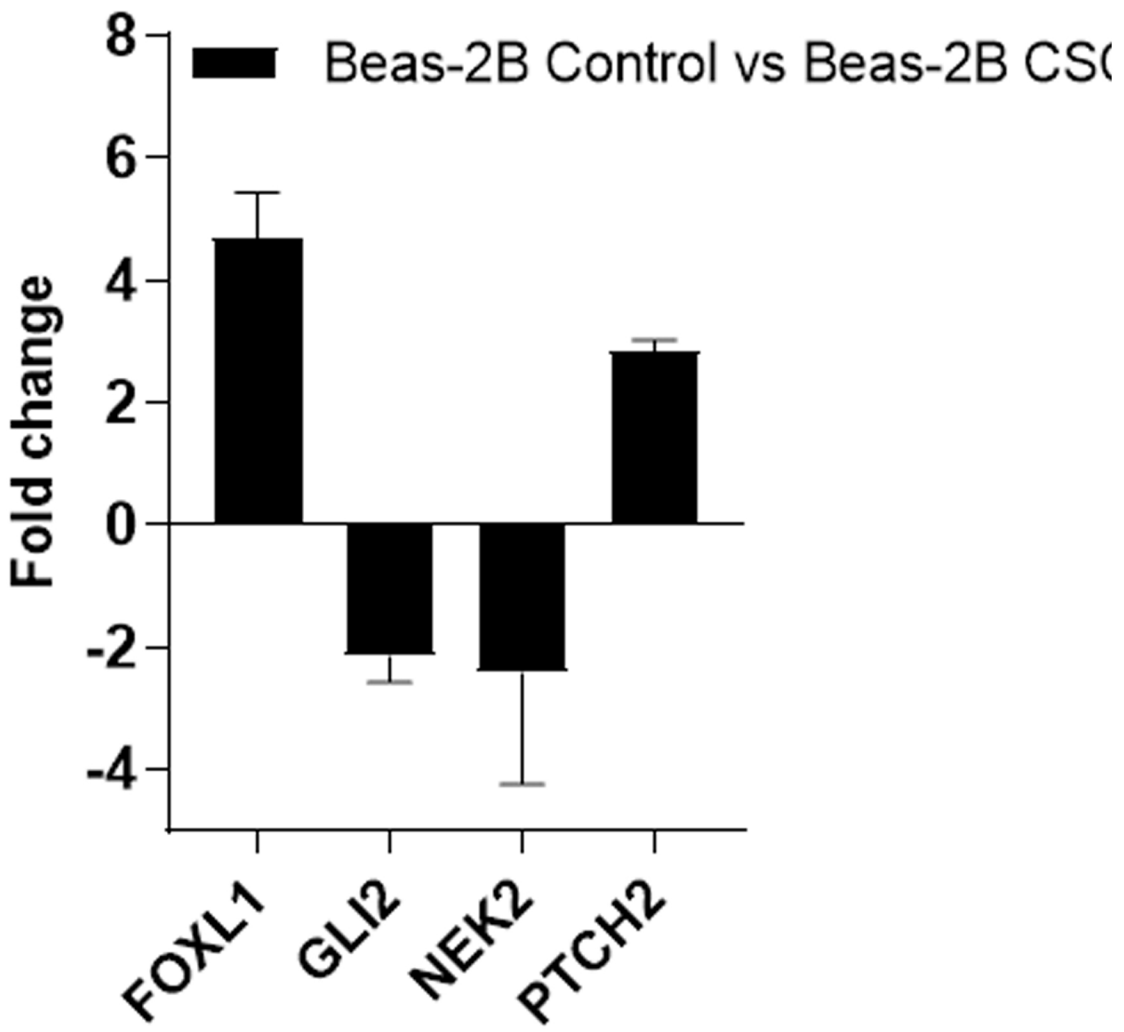

B

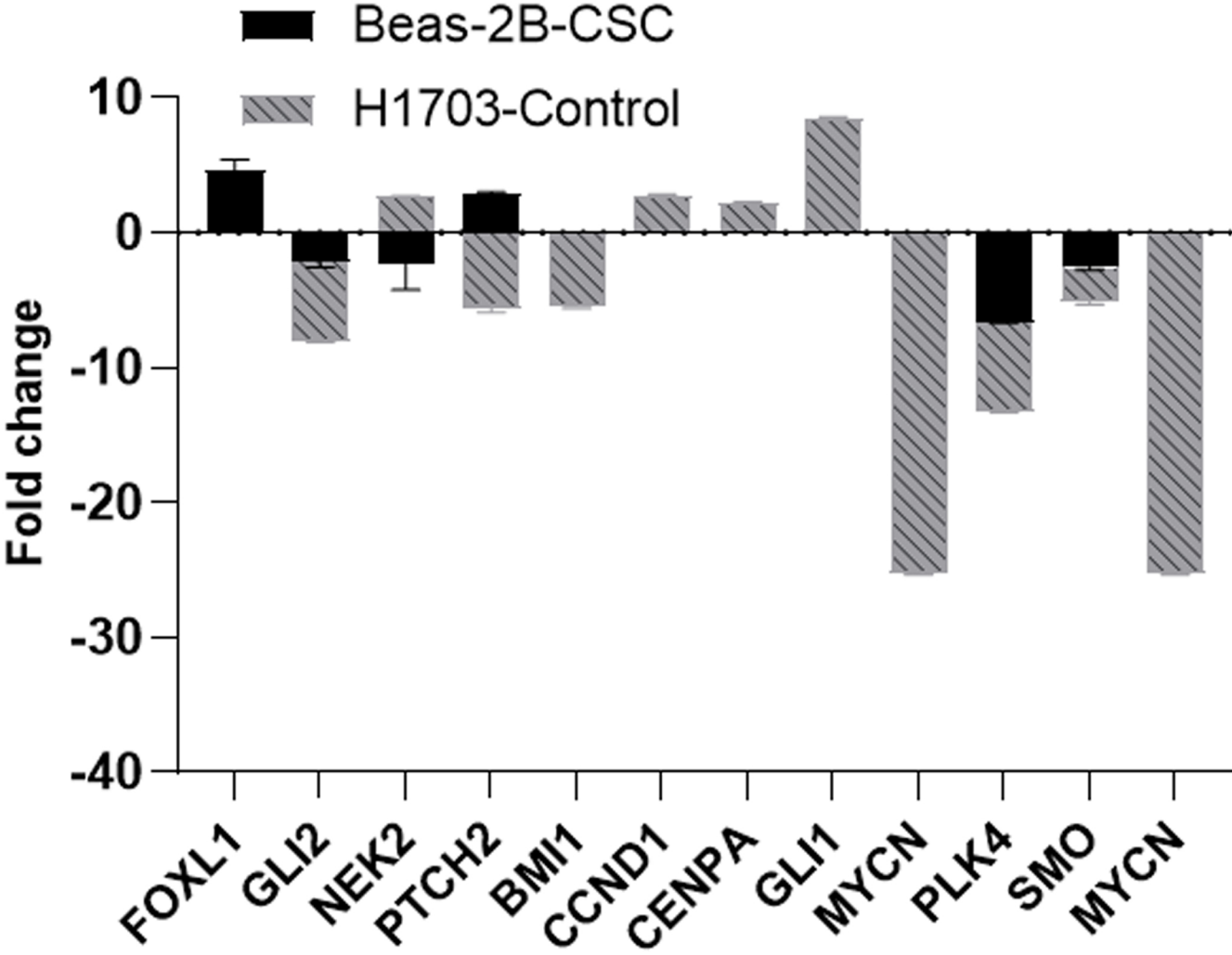

C

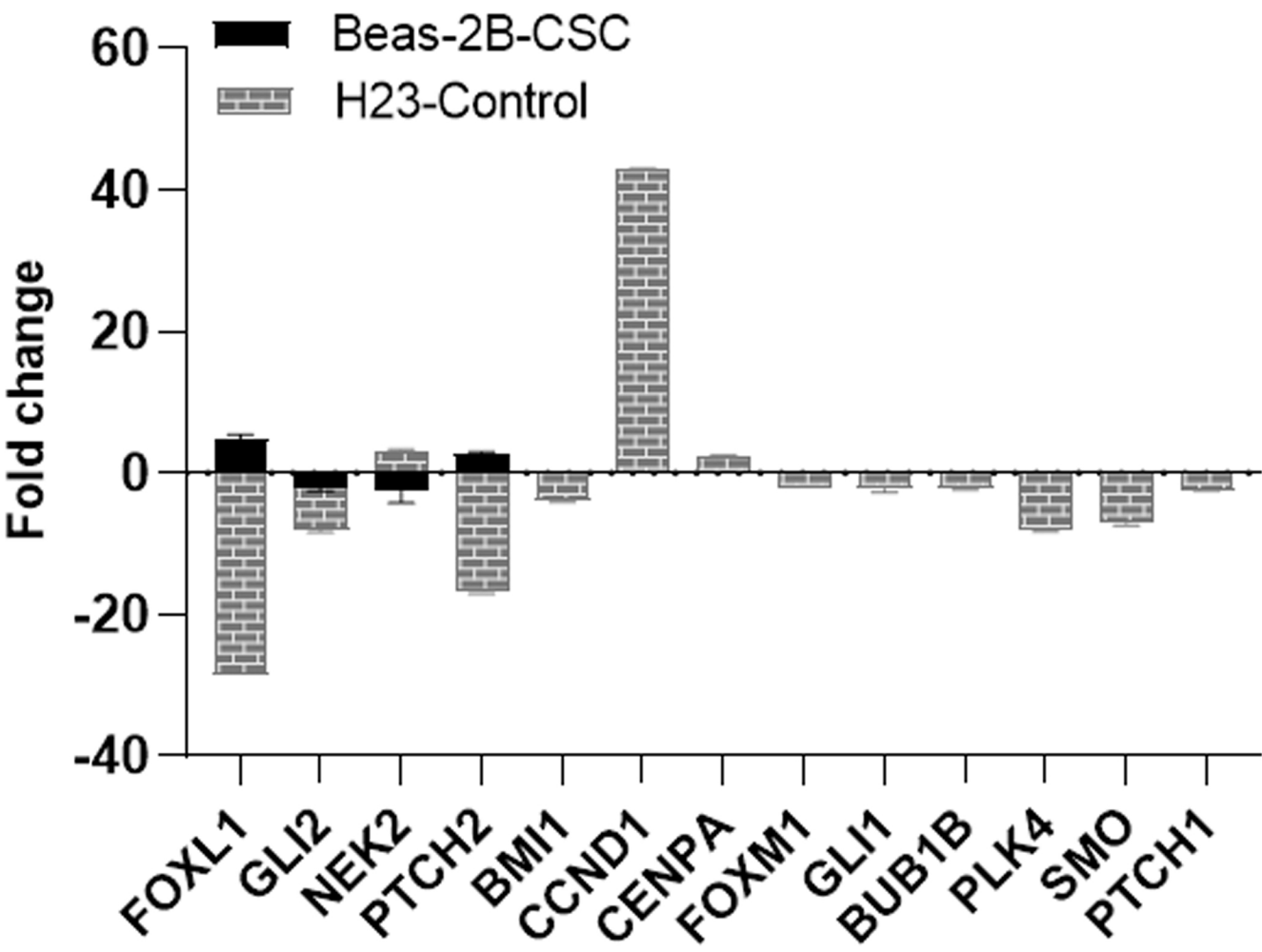

D

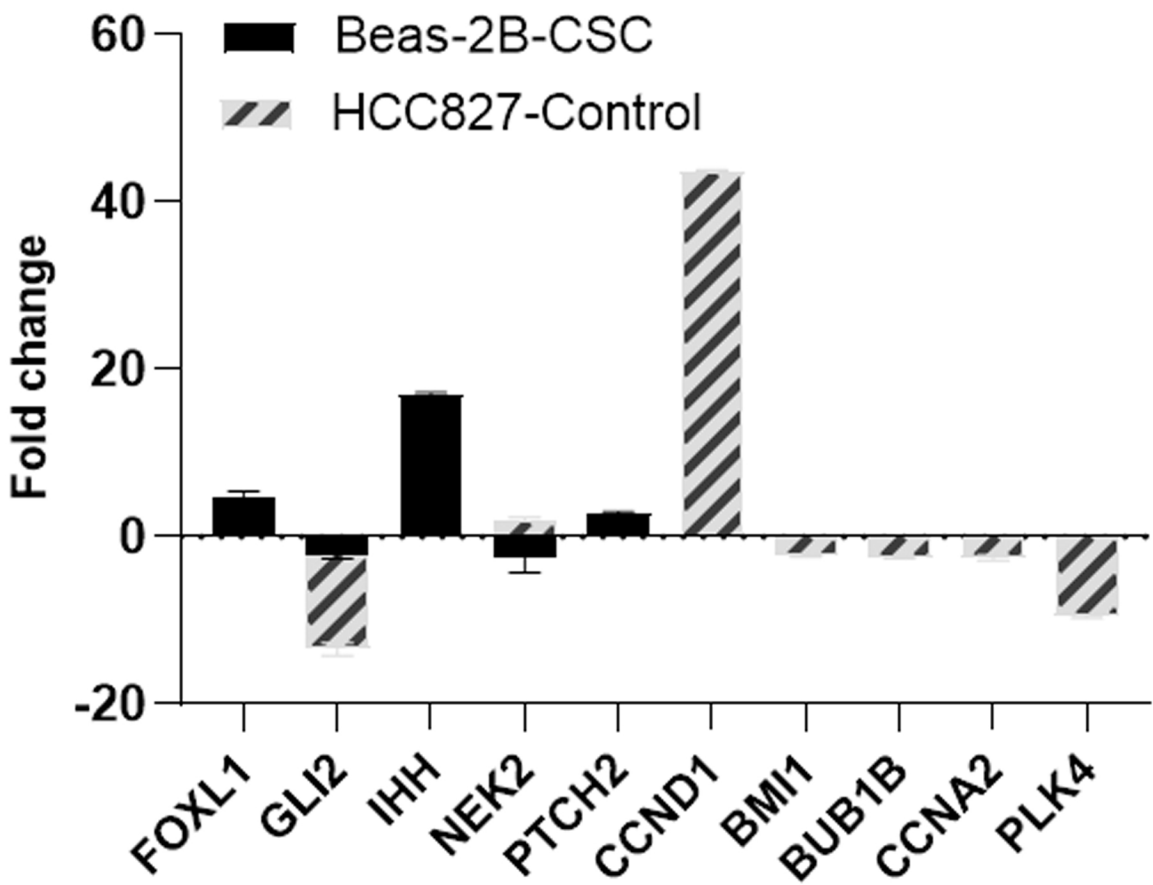

Supplement: Supplementary file 2 — Figure S2 [file CAM4-12-8499-s001.pdf]
